# Bilateral Neck Pyomyositis Caused by* Staphylococcus capitis* and* Staphylococcus saccharolyticus* in a Diabetic Adult

**DOI:** 10.1155/2017/3713212

**Published:** 2017-10-04

**Authors:** Nicholas Young, Hasan Bhally

**Affiliations:** Department of Medicine and Infectious Diseases, North Shore Hospital, Private Bag 93503, Auckland 0620, New Zealand

## Abstract

We report a case of pyomyositis of the paraspinal neck muscles caused by two coagulase-negative staphylococci:* Staphylococcus capitis *and* Staphylococcus saccharolyticus. *Inflammation in the spermatic cords was an additional feature of this infection. Treatment with six weeks of first-generation cephalosporin therapy resulted in complete clinical and radiological resolution.

## 1. Introduction

Coagulase-negative staphylococci (CoNS), with over 40 recognised species or subspecies, are normal inhabitants of human skin. Infections caused by CoNS are well recognised, including bacteraemia from* Staphylococcus epidermidis* associated with indwelling medical devices, urinary tract infections in young females from* Staphylococcus saprophyticus*, and musculoskeletal infections or endocarditis from* Staphylococcus lugdunensis *[1].

Despite the range of infections caused by CoNS, pyomyositis (a bacterial infection of skeletal muscle most commonly caused by* Staphylococcus aureus*) is exceedingly rare. To the best of our knowledge, only two cases of pyomyositis caused by CoNS have been reported, both due to* S. epidermidis*. Both cases presented with unilateral pyomyositis involving the thigh and semimembranosus muscles, respectively, and occurred in immunocompetent patients [2, 3].

Here, we report the first case of bilateral pyomyositis of the paraspinal neck muscles caused by* Staphylococcus capitis *and* Staphylococcus saccharolyticus* coinfection, which was successfully treated with six weeks of first-generation cephalosporin therapy.

## 2. Case Report

A 48-year-old Caucasian man was admitted to hospital with four weeks of atraumatic, progressive pain in the posterior neck. Three days prior to presentation, this pain became excruciating, and neck swelling, fever, and bilateral groin pain developed. He had no rash, joint pain, weight loss, dysuria, or urethral discharge. There was no recent history of skin or soft tissue infections, respiratory tract infection, dermatological conditions, or intravenous drug use.

He was known to have type 2 diabetes mellitus, which was well controlled on metformin with glycated haemoglobin of 49 mmol/mol, and hyperlipidaemia treated with atorvastatin 40 mg daily for the last two years.

On presentation, he was febrile at 38.5°C. Bilateral tenderness and swelling of the posterior neck muscles were found, with no overlying skin erythema. Range of motion of the neck was restricted due to pain. Tenderness in the inguinal area was noted bilaterally, with no palpable abnormalities. Scrotal examination was normal. Blood investigations revealed a C-reactive protein of 372 milligrams per litre (normal < 5) with neutrophilia of 16.2 × 10^9^ per litre (normal 1.90–7.50 × 10^9^). Creatinine kinase was normal at 38 units per litre. Aldolase was not measured. ANCA, ANA, and HIV were negative. Thyroid function was normal. Multiple peripheral blood cultures were sterile. Midstream urine was sterile, with an inactive sediment.

**Figure 1:**
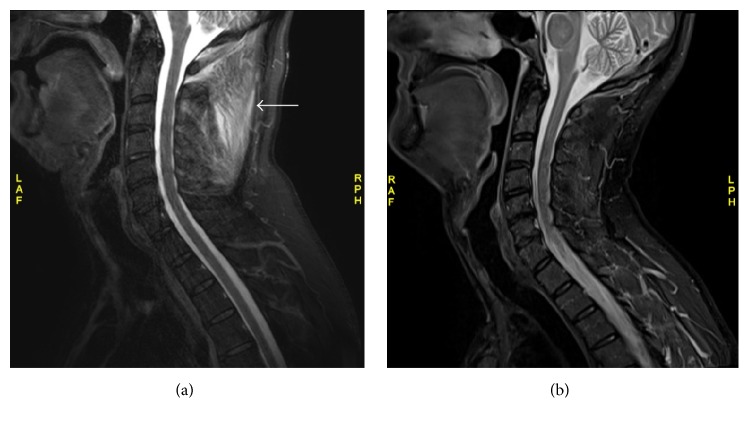
(a) T2-weighted sagittal MRI of neck pretreatment, showing hyperintensity in the posterior paraspinal muscles, consistent with myositis (arrow). (b) T2-weighted sagittal MRI of neck following six weeks of antibiotic treatment, showing complete resolution of myositis.

Magnetic resonance imaging of the neck demonstrated diffuse T2 hyperintensity in the posterior paraspinal muscles and to a lesser extent the sternocleidomastoid and trapezius muscles, consistent with myositis (Figure 1(a)). Ultrasonography of the groin showed increased echogenicity of both spermatic cords, consistent with inflammation. Computed tomography of the chest, abdomen, and pelvis was unremarkable, with no evidence of malignancy or lymphadenopathy.

Given the unclear aetiology of his presentation, an ultrasound guided muscle biopsy was performed, prior to the administration of any antibiotics. Multiple specimens were obtained through a fine needle aspirate and core needle biopsy of the left paravertebral and supraclavicular muscles. Growth of CoNS was detected on sheep blood agar and chocolate plates at 24 hours on all the tissue specimens, with two different colony morphologies. Species identification was performed through Bruker MALDI-TOF Biotyper (ver 3.1.66) with a high logarithmic score for* S. capitis* (1.95–2.21) and* S. saccharolyticus* (2.27–2.31) from each colony morphology. Further molecular characterization to determine strain relatedness was not able to be performed. All specimens were resistant to penicillin but susceptible to flucloxacillin. Histology showed oedema with mild neutrophilic inflammatory infiltrate.

During the one week of hospitalization prior to culture results becoming available, his neck pain had worsened despite opioid analgesia, with ongoing high fevers and increasing C-reactive protein. Antibiotic therapy with cefazolin 2 g intravenously every eight hours was therefore initiated, while acknowledging the possibility of skin contamination causing the positive culture results. He responded well, with resolution of fever, reduction in inflammatory markers, and slow but complete resolution of pain at the end of four weeks of treatment. An additional two weeks of oral cephalexin 500 mg four times per day was prescribed due to slow resolution of pain. Repeat magnetic resonance imaging demonstrated complete resolution of myositis (Figure 1(b)). The significance of the abnormality in the spermatic cords remains unclear, but this also normalized on repeat ultrasonography. No predisposing event that caused this patient to develop pyomyositis was found, although diabetes mellitus was a contributing factor.

## 3. Discussion

Pyomyositis is an acute bacterial infection of skeletal muscle. S*. aureus *is the causative pathogen in more than 75% of cases, followed by* S. pyogenes*, other beta-haemolytic streptococci*, Streptococcus pneumoniae, Streptococcus viridans, Escherichia coli,* and* Mycobacterium tuberculosis* [4]. CoNS are very rarely implicated.

Primary pyomyositis (pyomyositis in absence of infection of adjacent structures) is believed to be initiated by muscle seeding from transient bacteraemia. A predisposing muscle abnormality, such as trauma or haematoma, may provide bacteria with a favourable medium in which to reproduce. Immunosuppression is often present, including HIV infection, malignancy, rheumatologic conditions, and diabetes mellitus [4].

*S. capitis* is part of the normal skin flora, commonly found around the face, ears, and neck. Some strains of* S. capitis* contain the arginine catabolic mobile element (ACME), which is thought to improve the organism's ability to colonize the skin [5]. Although not previously reported as a cause of pyomyositis, it has been identified in other musculoskeletal infections (including osteomyelitis and prosthetic joint infection), endocarditis, meningitis, and neonatal sepsis [6].

*S. saccharolyticus *(formerly* Peptostreptococcus saccharolyticus*) is the only anaerobic CoNS and is also part of the normal skin flora, commonly found around the forehead [7]. It is rarely associated with disease, causing two cases of infective endocarditis [8, 9] and one case of thoracic spondylodiscitis [10], postsurgical septic arthritis of the glenohumeral joint [11], bone marrow infection [12], and pneumonia where the isolate was multidrug resistant [13]. It has also been isolated from contaminated platelet concentrates, although it has not been reported as a cause of transfusion-associated bacteraemia [14].

Certain CoNS, especially* S. epidermidis*,* S. lugdunensis,* and* S. haemolyticus,* have virulence factors that enhance their pathogenic potential.* S. epidermidis* can produce a structured, tenacious biofilm on the surface of indwelling devices consisting of polysaccharide intercellular adhesin (PIA), creating an environment that results in antibiotic tolerance and evasion of host defenses.* S. lugdunensis* possesses several virulence factors including a thermostable DNase clumping factor, an extracellular glycocalyx, lipase, haemolysin, and a fatty acid enzyme-modifying enzyme. Similar characteristics have not been reported for* S. capitis* or* S. saccharolyticus* [1].

The case described here is unique given the rarely pathogenic species of CoNS, the lack of indwelling devices as the portal of entry, coinfection with two species of CoNS, the bilateral distribution of myositis, and the unexplained spermatic cord inflammation. While we were not able to perform definitive molecular testing to confirm relatedness between the CoNS isolates from the two biopsy sites, the prolonged duration of symptoms preceding treatment, the multiple positive tissue cultures from two different biopsy sites, clinical and radiological improvement after initiation of antibiotics, and absence of an alternative diagnosis all support the pathogenic role of CoNS in this case. Idiopathic inflammatory myositis of the neck muscles has been described [15], although this felt unlikely given the patient's improvement despite not receiving immunosuppressive agents. We therefore conclude that* S. capitis *and* S. saccharolyticus *caused pyomyositis in this diabetic male. These CoNS should be considered pathogenic in similar clinical scenarios. Improved understanding of the pathogenesis of CoNS strains other than* S. epidermidis, S. lugdunensis, *and* S. haemolyticus, *including specific virulence factors and contributing host or bacterial strain characteristics, would be beneficial for clinicians to better recognise and manage such syndromes.
